# Simplified Optimal Estimation of Time-Varying Electromyogram Standard Deviation (EMGσ): Evaluation on Two Datasets

**DOI:** 10.3390/s21155165

**Published:** 2021-07-30

**Authors:** He Wang, Kiriaki J. Rajotte, Haopeng Wang, Chenyun Dai, Ziling Zhu, Xinming Huang, Edward A. Clancy

**Affiliations:** 1 Worcester Polytechnic Institute, Worcester, MA 01609, USA; hwang9@wpi.edu (H.W.); kjrajotte@wpi.edu (K.J.R.); hwang10@wpi.edu (H.W.); zzhu2@wpi.edu (Z.Z.); xhuang@wpi.edu (X.H.); 2 Center for Biomedical Engineering, Fudan University, Shanghai 200433, China; chenyundai@fudan.edu.cn

**Keywords:** biological system modeling, biomedical signal processing, electromyogram, electromyogram (EMG) amplitude estimation, electromyography, advanced signal processing

## Abstract

To facilitate the broader use of EMG signal whitening, we studied four whitening procedures of various complexities, as well as the roles of sampling rate and noise correction. We separately analyzed force-varying and constant-force contractions from 64 subjects who completed constant-posture tasks about the elbow over a range of forces from 0% to 50% maximum voluntary contraction (MVC). From the constant-force tasks, we found that noise correction via the root difference of squares (RDS) method consistently reduced EMG recording noise, often by a factor of 5–10. All other primary results were from the force-varying contractions. Sampling at 4096 Hz provided small and statistically significant improvements over sampling at 2048 Hz (~3%), which, in turn, provided small improvements over sampling at 1024 Hz (~4%). In comparing equivalent processing variants at a sampling rate of 4096 Hz, whitening filters calibrated to the EMG spectrum of each subject generally performed best (4.74% MVC EMG-force error), followed by one universal whitening filter for all subjects (4.83% MVC error), followed by a high-pass filter whitening method (4.89% MVC error) and then a first difference whitening filter (4.91% MVC error)—but none of these statistically differed. Each did significantly improve from EMG-force error without whitening (5.55% MVC). The first difference is an excellent whitening option over this range of contraction forces since no calibration or algorithm decisions are required.

## 1. Introduction

**Figure 1:**
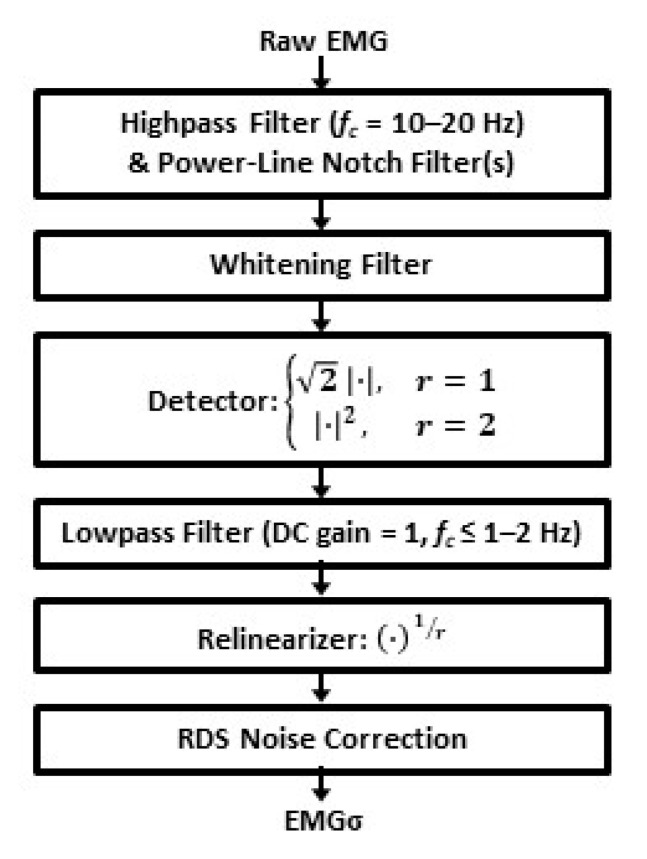
Cascade of filter steps used to produce an advanced EMGσ estimate. Exponent *r* equals 1 or 2. RDS = root difference of squares.

Estimates of the standard deviation of the surface electromyogram (EMG) signal (EMGσ) serve as a global measure of muscular activation [1,2,3]. EMGσ is used to estimate the torque [4,5,6,7,8,9] and mechanical impedance [10,11,12,13,14,15] of a joint in motor control research [16] and many applications [17] including prosthesis control [18,19,20], ergonomic assessment [21,22], and clinical biomechanics [23,24]. Advanced single-channel EMGσ estimates comprise a cascade of (Figure 1) [25] a high-pass filter at 10–20 Hz (to remove DC offsets and attenuate motion artifacts; the precise cut-off selection is not critical over this range), notch filters (to reject power-line interference and its harmonics), a whitening filter (to temporally uncorrelate the samples), a detector (absolute value or square; when absolute value detection is used, the data stream should be multiplied by $\sqrt{2}$ for the proper scaling of the subsequent resting noise correction [26]), a low-pass filter (*f_c_* ≤ a few Hz, DC gain = 1), a re-linearizer (only required if a square-law detector is used and would then consist of a square root operation), and resting noise correction. This final stage is realized via RDS processing [26] (the square root of the difference between the square of the processed EMG and the variance of noise estimate).

The whitening stage is *not* commonly utilized, likely because it can be too complex to implement and reduces the variability of EMGσ but does not alter its average value [27,28,29]. However, this reduction in variation is considerable—depending on the low-pass filter cut-off frequency [30], whitening can increase the signal-to-noise ratio (SNR) of constant effort contractions by 32–64% [29,31]. Applications that use EMGσ benefit in turn [4,10,32,33]. Hence, it is important to find simple and robust methods to implement whitening.

Regarding the complexity of whitening, Kaiser and Peterson [34] (see also [35]) adaptively varied the bandwidth of their analog whitening filter in an ad hoc manner to enable a larger frequency range during higher contraction levels, wherein the EMG signal power remained above that of the noise over a wider frequency span. Clancy and Farry [27] formalized this signal vs. noise trade-off as a function of frequency by cascading stages comprising (1) a fixed whitening filter, (2) an adaptive Wiener filter (an optimal linear filter when signal and noise are added), and (3) adaptive gain correction. Their filters were calibrated to each subject using active and rest contractions, a requirement that can be burdensome. Recently, a fixed bandwidth-limiting filter was added as Stage 4 [36].

**Figure 2:**
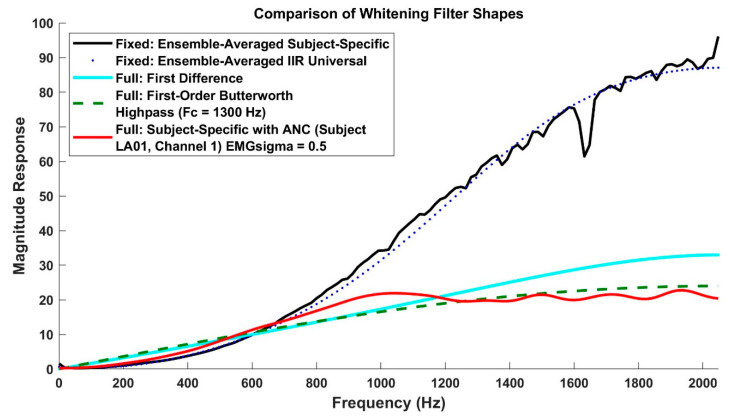
Comparison of whitening filter shapes for filters with a sampling rate of 4096 Hz. The gain of first difference and high-pass filters were manually matched over the frequency range up to ~1000 Hz, the region containing most EMG power. The graph based on ensemble filter shapes only shows the fixed Stage 1 filter for the subject-specific method and, separately, the Universal IIR method. ANC = adaptive noise canceling.

Potvin and Brown [37] demonstrated simplified whitening by using a first-order Butterworth high-pass filter with a cut-off frequency of 410 Hz (same filter for all subjects), and inherently limited whitening out to a frequency of 500 Hz (due to their 1000 Hz sampling rate). As shown in Figure 2, the low order and high cut-off frequency of such filters cause their magnitude response to closely resemble the shape of a whitening filter, at least up to 500–600 Hz in frequency. The filter has low gain at low frequencies and high gain at high frequencies, thus closely matching the inverse of the magnitude spectrum of EMG, as required for whitening [29,31,38], and it certainly does not exhibit the shape of an ideal high-pass filter. Others [39,40] have used the first difference of the EMG signal (sampled at ~1000 Hz) to achieve simplified whitening. A first difference also has a shape similar to a whitening filter (Figure 2). The limited bandwidth of each of these methods is important, since applying gain to higher signal frequencies—wherein the spectrum can have more noise than signal—leads to the amplification of this noise and the degradation of EMGσ performance [27,35,36].

The use of the resting noise correction stage is somewhat uncommon in academic research, but it is common in applications involving low-level contraction or rest. For example, in the absence of noise correction, the pose of myoelectrically-controlled exoskeletons, orthoses, and prosthesis [41] would drift during rest (since the resting EMG signal is not null-valued). In research settings, noise correction is more common when studying low-level muscular activity [42], since rest noise is additive and larger (in relation to EMGσ) during such contractions. A reason for the lack of use of this important stage may be that a calibration of noise variance is required.

**Figure 3:**
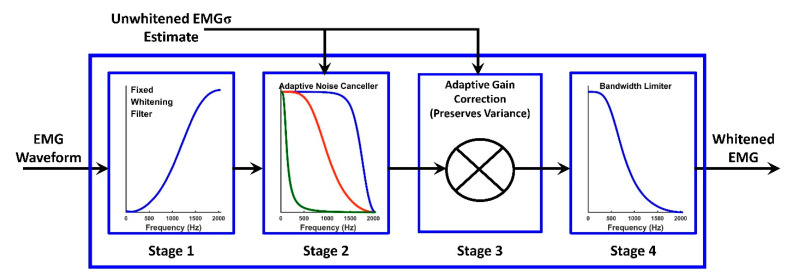
Four-stage whitening filter [27,36]. Stage 1 is a fixed linear filter that whitens the true EMG signal. Stage 2 is an adaptive linear low-pass filter (formally a Wiener filter) that progressively rejects more of the higher frequencies as the effort level decreases. Stage 3 is an adaptive gain that preserves the variance of the true EMG through the first three stages. Stage 4 is a high-order, fixed low-pass filter that limits whitening bandwidth.

Inspired by the simple whitening techniques of first-order high-pass filtering and first differencing, we studied simplified designs of optimal whitening filters, particularly the fixed whitening stage (Stage 1 in Figure 3). Since EMG spectral shape/statistical bandwidth (which determines whitening filter shape) is known to somewhat vary with electrode size and shape [29], there is some shape variation as these factors change. However, many applications fix their electrode topology (e.g., encased electrode-amplifiers). Thus, these factors are also fixed for that application. Therefore, a universal fixed whitening stage shape might be acceptable for such applications.

We developed a universal fixed whitening stage (i.e., not calibrated to each individual subject) and tested its performance against that of the two simplified whitening techniques on two datasets (collected within the same experimental sessions) of constant-posture contractions about the elbow. Our first dataset comprised force-varying contractions, ranging over 0–50% of maximum voluntary contraction (MVC). This dataset provides an excellent evaluation of whitening algorithms. However, because of the high average level of contraction, we anticipated this dataset to be a poor indicator of the influence of additive and high-frequency noise that can adversely affect whitening [35], as well as the performance of the noise correction stage [43]. Thus, our second dataset comprised constant-force contractions at 0% and, separately, 50% MVC. EMG from the 0% MVCs entirely comprised additive background noise, while EMGs from 50% MVCs had very low relative noise power. However, both 0% and 50% MVC EMG should be improved by whitening. A preliminary report of this work appeared in [43].

## 2. Experimental Data and Universal Whitening Filters

### 2.1. Experimental Data

The WPI Institutional Review Board exempted from supervision analysis of de-identified data from 64 able-bodied subjects, acquired during four prior studies with overlapping protocols [4,27,44,45]. A subject was seated and secured to an experimental chair with their right shoulder abducted 90°, their elbow flexed 90°, and their hand supinated perpendicular to the floor. Their wrist was cuffed to a load cell that measured constant-posture elbow torque (see [4]; Figure 1). Skin above the triceps and biceps muscles was scrubbed with an alcohol wipe, and (in the two latter studies) gel was applied. Four bipolar EMG electrode-amplifiers were secured over each of the triceps and biceps muscles, midway between the elbow and the midpoint of the upper arm, in a tightly-spaced circumferential row centered on the muscle mid-line. Each electrode-amplifier had stainless steel, hemispherical contacts of 4 or 8 mm in diameter, separated 10 mm edge-to-edge, and oriented along the muscle’s long axis. A reference electrode was secured next to the active electrodes. Each EMG channel had a CMRR ≥ 90 dB at 60 Hz, selectable gain, a 10 or 15 Hz high-pass filter (second or fourth order), and a 1800 or 2000 Hz low-pass filter (fourth order). Load cell and EMG data were sampled at 4096 Hz (16-bits). Achieved force was fed back on a real-time computer display, along with a force target.

After a short warm-up, elbow flexion and (separately) extension MVC forces were measured without using force feedback. At least 20–30 min elapsed between the time at which the electrodes were mounted and the completion of these MVC measurements. Data for the first dataset were collected as three, 30 s duration, force-varying contractions. Subjects tracked a computer target that queued a 1 Hz bandwidth, uniformly random force target spanning 50% MVC extension to 50% MVC flexion. Constant-force data for the second dataset were collected at 50% MVC extension, 50% MVC flexion (using force feedback), and 0% MVC (i.e., arm at rest, removed from the wrist cuff) for 5 s each. Two recordings for each condition were acquired. A rest of 2–3 min was provided between contraction trials to avoid cumulative fatigue.

### 2.2. Development of Universal Whitening Filters

Traditionally, *subject-specific* whitening filters are calibrated for each electrode of each subject based on the power spectral density (PSD) of a 0% MVC and an active MVC (e.g., 50%), resulting in the processing shown in Figure 3 [27,36]. Stage 1 is a fixed linear filter that whitens the spectrum of the noise-free (“true”) 50% MVC EMG. Its magnitude response is found via spectral subtraction as: [PSD50−PSD0]−12, where $PSD_{x}$ is the EMG PSD at *x%* MVC (Welch method, Hamming window, 50% overlap, and 2048-point discrete Fourier transform). Thus, the Stage 1 filter has high gain at frequencies wherein there is low EMG power and vice versa [28,31,34,46]. Unfortunately, this filter shape also accentuates the higher noise frequencies, particularly during low-effort contractions. Thus, Stage 2 is an adaptive linear low-pass filter (formally a Wiener filter) that progressively attenuates higher frequencies as the effort level decreases [27,34]. Stage 3 applies an adaptive gain such that the variance of the true EMG signal is preserved through the first three stages. (The original development of this adaptive gain stage (Stage 3) preserved the variance of the entire input signal—since the input signal comprises noise plus the true EMG signal, we improved the algorithm to only preserve the variance of the true EMG signal portion.) These three cascaded linear systems are realized as a single adaptive filter stage comprising 100 distinct 60th-order FIR filters, providing adaptation over the range from rest to MVC with an increment (adaptation resolution) of 1% MVC. Adaptation is based on the current unwhitened EMGσ estimate. Stage 4 is a high-order, fixed low-pass filter (*f_c_* ≥ 600 Hz) that limits whitening bandwidth to further attenuate high frequency noise.

For our efficient *universal* first-stage IIR whitening filter, we started by determining the desired Stage 1 magnitude response for each electrode from each subject via the subject-specific method [27]. These magnitudes were then ensemble-averaged across the 512 available responses (64 subjects × 8 electrodes/subject), forming the desired universal magnitude response. Using this desired response, we developed a second-order IIR Stage 1 (fixed) whitening filter (thus, *D* = 5 free parameters) using the novel filter design method of differential evolution [47,48]. A second-order IIR filter is far more computationally efficient to implement than the 60th-order filter implementation of [27]. Briefly, this method is initialized by random instantiation (all randomization used independent uniform distributions) of *P* parameter vectors ${\underline{x}}_{p,G}$ (each of length *D*), where p={1, 2, 3, ⋯,P} indexes the *P* vectors and *G* indexes the generation (i.e., optimization process iteration). The three feedforward coefficients ranged over [–100, 100], and the two feedback coefficients ranged over [–1, 1]. After initialization, this approach uses a three-step, parallel, direct search. First, each parameter of the *P* candidate vectors is mutated to produce next generation parameters as:

(1) $$v_{d,p,G+1}=x_{d,p,G}+K\;\left(x_{d,r1,G}-x_{d,p,G}\right)+F\;\left(x_{d,r2,G}-x_{d,r3,G}\right),$$

where d={1, 2, 3, 4, 5} indexes the *D* parameters within a vector, and {r1, r2, r3} are selected randomly over the integer range [1, *P*] without replacement. Scaling factor *F* and combination factor *K* are real-valued over the range [0, 2]. Second, to increase parameter diversity, crossover is applied to each parameter within each mutated vector to form *P* “trial” vectors, ${\underline{\mu }}_{p,G+1}$, as:

(2) $$\mu_{d,p,G+1}=\{\begin{array}{cc} v_{d,p,G+1}, & R_{d,p,G+1}\leq CR\;\mathrm{or}\;d=I_{d,p,G+1}\\ x_{d,p,G}, & \mathrm{otherwise} \end{array},$$

where $0<R_{d,p,G+1}<1$ is a random number, $1\leq I_{d,p,G+1}\leq D$ is a randomly chosen parameter index, and 0<CR<1 is a user-set crossover constant. The indicated randomizations are performed for each parameter within each vector, but vectors remain unchanged if the resultant filter is unstable (i.e., poles outside the unit circle). Third, *selection* consists of comparing the performance of each trial vector ${\underline{\mu }}_{p,G+1}$ to its respective parent vector ${\underline{x}}_{p,G+1}$ and then retaining the better vector. We compared weighted RMS error of the achieved magnitude response to the desired magnitude response. For the 4096 and 2048 Hz sampling rates, a weight of 0.8 was applied to frequencies of ≤600 Hz and a weight of 0.2 to frequencies of >600 Hz, because a whitening band limit of 600 Hz is common [36]. For the 1024 Hz sampling rate, a frequency cut-off of ≤300 Hz was empirically selected. We found [49] that errors are fully stabilized by *G* = 280 generations when using *K* = *F* = 0.2 and *CR* = 0.5. The best of the resulting *P* vectors was chosen to be the solution vector. The resulting second-order IIR filter is shown as the Stage 1 magnitude response in Figure 3 (see also Figure 2). For reference, Table A1 in Appendix A gives the complete set of universal first-stage IIR filter coefficients for all sampling rates.

## 3. Study 1: Force-Varying Contractions

### 3.1. Methods of Analysis—Study 1

Off-line (using MATLAB), each EMG channel for each trial was (Figure 1) normalized to a 50% MVC contraction (using EMG from that channel), high-pass filtered (15 Hz cut-off, fourth-order Butterworth), second-order IIR notch filtered at 60 Hz and its harmonics, optionally whitened and band-limited, rectified (and multiplied by $\sqrt{2}$), low-pass filtered (16 Hz cut-off, ninth-order Chebyshev Type I filter; 0.05 dB peak-to-peak passband ripple), and then optionally noise corrected. Note that further low-pass filtering was inherently provided by the subsequent EMGσ-force processing [50]. When used, noise correction was implemented using RDS processing [26], with the noise variance estimated from a 0% MVC contraction after the whitening stages.

The four extension and four flexion EMGσ estimates (as well as the squares of these values), as well as the measured torque, from two trials per subject were used to train an EMGσ-force model via regression (i.e., a 16-input, 1-output model). This model was 15th-order quadratic FIR per channel that was fit using the Moore–Penrose inverse in which singular values were discarded if their ratio to the largest singular value was less than 0.0056 [4]. The RMS error between the actual torque from the third (test) trial and that estimated by the EMGσ-force model (omitting the first 500 ms to account for startup transients) was used to assess performance. The mean ± standard deviation RMS error (RMSE), across the 64 subjects, is reported.

Performance was compared for all combinations of the following:

- Sampling rate: We used the original 4096 Hz sampling rate and rates of 2048 and 1024 Hz, representing a range of rates used in practice. Sampling rates are important to study, since high sampling rates are particularly sensitive to whitening-induced noise at higher frequencies [27,36]. When decimated, the signal was low-pass filtered at 80% of the new Nyquist rate (seventh-order Chebyshev Type I filter, 0.05 dB peak-to-peak passband ripple) and then down-sampled.
- Stage 1–3 whitening filters: We studied six variations: (1, 2) our traditional subject-specific 60th-order FIR Stage 1 whitening filter with and without Stages 2–3, (3, 4) second-order universal IIR Stage 1 whitening with and without Stages 2–3, (5) high-pass filter whitening without Stages 2–3, and (6) first difference whitening without Stages 2–3. The high-pass and first difference methods were studied without Stages 2–3, since these stages are not part of these methods. For high-pass filter whitening, the cut-off frequency was varied upwards from 100 Hz in increments of 30 Hz. The maximum cut-off frequency was the lesser of 1930 Hz or the value prior to exceeding the Nyquist frequency.
- Stage 4 whitening bandwidth: At sampling rates of 2048 and 4096 Hz, we varied the whitening bandwidth to 1000 and 600 Hz via ninth-order, Chebyshev Type I, 0.05 dB peak-to-peak ripple low-pass filters that were causally implemented. Additionally, all sampling rates studied the full Nyquist frequency (no bandwidth limit).
- Noise correction: This was done with and without RDS processing.

Because this analysis was multivariate and Shapiro–Wilk tests found the Study 1 and Study 2 result data to be non-Gaussian, statistical comparisons between more than two groups were tested using a Friedman test. Pair-wise statistical comparisons used the Wilcoxon signed-rank test (with Bonferroni–Holm adjustment for multiple comparisons). A significance level of *p* < 0.05 was used. When listed, *p*-values are Bonferroni-adjusted.

### 3.2. Results—Study 1

**Figure 4:**
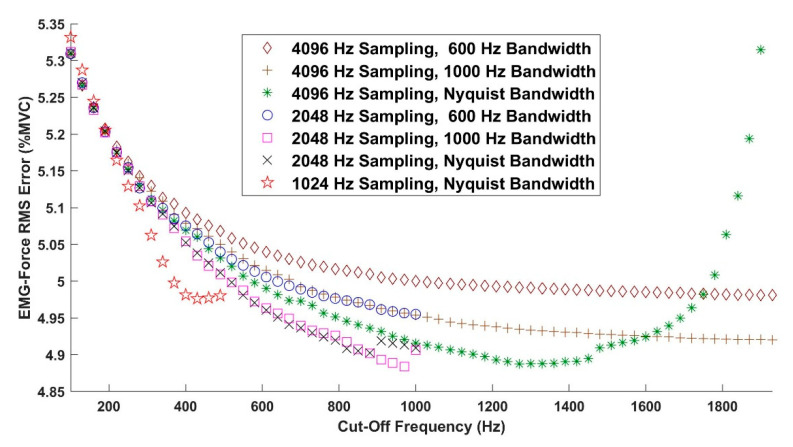
Mean EMG-force error results for first-order Butterworth high-pass filter whitening, as a function of high-pass filter cut-off frequency, for all processors including RDS processing. Standard deviations (not shown) each ranged from 2.04 to 2.58% MVC. Results without RDS processing were similar.

Figure 4 shows the mean EMG-force results when using high-pass filter whitening, as a function of high-pass filter cut-off frequency, for all of the processors including RDS processing. Standard deviations (not shown) each ranged from 2.04 to 2.58% MVC. Results without RDS processing were very similar (see statistical results below). For each high-pass whitening case, the results from the cut-off frequency yielding the lowest average error were used thereafter. When the signal was not bandwidth limited, a local minimum existed. Otherwise, the minimum tended to occur at the highest frequency.

**Figure 5:**
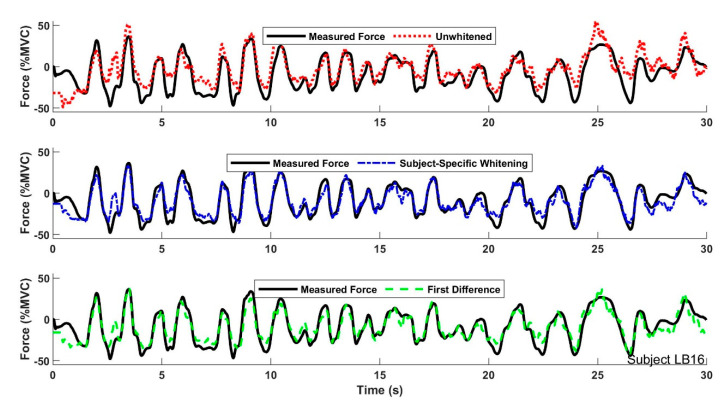
Example time-series plots from one trial showing measured force and EMG-force estimates using representative whitening variations. Each EMG-force processor used data sampled at 4096 Hz and RDS noise correction. The subject-specific whitener used adaptive noise canceling and a whitening bandwidth of 600 Hz. The first difference whitener used a whitening bandwidth of 600 Hz.

With these high-pass whitening results consolidated, Table 1 lists the complete set of summary results for Study 1 for all processors including RDS processing. Figure 5 shows an example time-series of the measured force during a trial and EMG-force estimators from three representative whitening variations. Note that EMG-force estimation without whitening shows the largest deviations from the measured force. Our statistical evaluation began along the RDS dimension by performing the 42 paired comparisons (RDS vs. no RDS) while fixing all other processing combinations. Only one statistical difference was found: a poorer performance was found without RDS processing when using the IIR whitening filter, without Stages 2–3 adaptive noise canceling (ANC), at the 4096 Hz sampling rate, with no whitening bandwidth-limiting filter ($p=10^{-5}$). Nonetheless, this difference in means was still only 0.4% MVC. Hence, only processors with RDS processing are listed in our results or were further analyzed statistically. Note that, without any whitening but with RDS processing, the mean ± standard deviation “baseline” EMG-force performance was 5.55 ± 2.4% MVC for all sampling rates. For comparison, whitening (compared to not whitening) always provided a statistically significant reduction in % MVC error, except when using RDS processing without ANC and without bandwidth limiting, for each of the subject-specific and IIR whitening methods.

We next statistically compared the six whitening variations (itemized above) separately for each combination of sampling rate and within each whitening band limit. A Friedman test checked for any differences across the six variations, and if any were found, Wilcoxon tests were used to evaluate each pair. At the 4096 Hz sampling rate, the first difference using a 1000 Hz bandwidth performed statistically poorer than high-pass. All other statistical differences at this sampling rate only involved the Nyquist bandwidth; (1) subject-specific without ANC performed poorer than all other whiteners; (2) IIR without ANC performed poorer than IIR with ANC, high-pass, and first difference, and (3) each of first difference, high-pass, and IIR without ANC whiteners performed poorer than subject-specific with ANC. At the 2048 Hz sampling rate, only first difference using a 600 Hz bandwidth performed poorer than high-pass. At the 1024 Hz sampling rate, no statistical differences were found. Of these statistical differences, the only difference that was both statistically significant and indicated a large change in % MVC occurred at the 4096 Hz sampling rate, when comparing processing without bandwidth limitation in which subject-specific processing performed noticeably poorer. This result is consistent with the low EMG SNR at high frequencies, which originally motivated the use of ANC [27,35].

Thereafter, we statistically compared across sampling rates for each condition (i.e., across each row of Table 1). When only two sampling rates were tested, we directly applied the Wilcoxon test; when three sampling rates were available, we applied the Friedman test, followed by post-hoc Wilcoxon tests (when the Friedman test found differences). The 4096 Hz sampling rate performed statistically poorer than the 2048 Hz sampling rate when comparing: (1) subject-specific without ANC using the Nyquist bandlimit, (2) IIR without ANC using the Nyquist bandlimit, and (3) high-pass using the 1000 Hz bandlimit; and the 4096 Hz sampling rate performed poorer than the 1024 Hz sampling rate for subject-specific without ANC using the Nyquist bandlimit. The 2048 Hz sampling rate performed statistically poorer than the 4096 Hz sampling rate when comparing: (1) subject-specific with ANC using any bandwidth, (2) IIR without ANC using the 600 Hz bandwidth, (3) IIR with ANC using the 600 and 1000 Hz bandwidths, and (4) first difference using the 1000 Hz or Nyquist bandwidths. The 1024 Hz sampling rate performed statistically poorer than the 2048 Hz sampling rate in all cases, except for subject-specific without ANC using the Nyquist bandwidth; and the 1024 Hz sampling rate performed poorer than the 4096 Hz sampling rate when only using the Nyquist bandwidth and subject-specific with ANC, IIR with ANC, and first difference. Overall, the 1024 Hz sampling rate generally performed poorer than the other rates. Though we itemized several statistical differences between the 4096 and 2048 Hz sampling rates, none of the strengths of these differences exceeded 0.11% MVC whenever bandwidth limiting was used.

Then, for subject-specific and IIR whiteners, we compared the performance of processors only using RDS processing vs. those only using ANC separately for each combination of sampling rate and whitening band limit. These paired evaluations each used the Wilcoxon signed-rank test. Four (of 14) separate tests were statistically significant, each showing adaptive whitening to perform better: subject-specific at the 4096 Hz sampling rate and 1000 Hz/Nyquist band limit, IIR at the 4096 Hz sampling rate and Nyquist band limit, and subject-specific at the 2048 Hz sampling rate and 1000 Hz band limit.

## 4. Study 2: Constant-Force Contractions

The force-varying contractions of Study 1 provided for an excellent evaluation of whitening methods and sampling rates, though only during the studied high contraction levels (effort spanned 50% MVC extension to 50% MVC flexion but never remained near 0% MVC). These high contraction levels obscure the influence of noise, which is more prevalent at lower contraction levels, and thus the impact of RDS processing [26]. However, much applied use of processed EMG includes extensive periods of rest and low effort levels (e.g., [51]). Thus, Study 2 explicitly studied EMG processing performance during the lowest possible contraction level of 0% MVC (rest). Of course, evaluating constant-force rest contractions in isolation would not be informative, since a processor that indiscriminately set all processed values to zero would artifactually appear optimal. Thus, we studied the ratio of a rest contraction to a 50% MVC, with the EMG from both undergoing the same processing. The lower the ratio, the better the separation of signal from noise.

### 4.1. Methods of Analysis—Study 2

EMGσ processing for the 5 s duration constant-force trials was identical to that of Study 1 (including use of the best high-pass whitening filter cut-off frequencies listed in Table 1), except that the low-pass filter stage consisted of a 200 ms moving average (which is more appropriate for constant-force EMGσ processing [28,29]). Only the four triceps electrodes were processed from the two 50% MVC extension trials, and only the four biceps electrodes were processed from the two 50% MVC flexion trials. The corresponding electrodes were processed from the two rest trials. The first 200 ms were omitted from each processed EMGσ to account for startup transients. The first trial from each contraction level was used for calibration (i.e., normalization to 50% MVC and to form subject-specific whitening filters when utilized) and the second was used for testing. Then, for cross-validation, the calibration and testing sets were exchanged.

For evaluation, the average value of each EMGσ at 0% MVC was divided by the average EMGσ from the same electrode at 50% MVC. The average ratio from the two cross-validation folds was used as the result for each subject and electrode, as well as in the statistical comparisons. A lower ratio denotes better noise attenuation performance. As in Study 1, the complete evaluation was repeated for the same combinations of sampling rates, Stage 1–3 whitening filter variations, up to three Stage 4 whitening bandwidth limits, and with vs. without RDS processing. Statistical analysis proceeded in the same manner as was used for Study 1.

### 4.2. Results—Study 2

**Figure 6:**
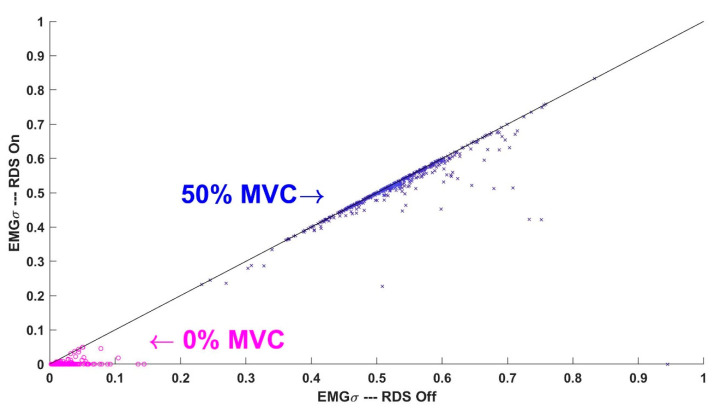
Scatter plot of the 512 EMGσ values (64 subjects × 8 electrodes/subject) with vs. without RDS processing for each of 0% (magenta circles) and 50% (blue x’s) MVC trials. Results correspond to processing with subject-specific whitening adaptive noise canceling, no bandwidth limiting, and a 4096 Hz sampling rate. The clusters of 0% and 50% MVC values do not overlap. The solid black line is the line of agreement.

Table 2 shows the summary results from all ratio tests. Figure 6 shows a scatter plot of the 512 EMGσ values (64 subjects × 8 electrodes/subject) with vs. without RDS processing, for each of the 0% and 50% MVC trials when using the most advanced processing—subject-specific whitening with ANC, no bandwidth limiting, and a 4096 Hz sampling rate. As expected, RDS processing had limited influence on EMGσ values at 50% MVC (most points fall on the line of agreement), but it had the desirable effect of setting most 0% MVC EMGσ values to/near zero [26]. Our statistical evaluation again began along the RDS dimension, performing the 42 paired comparisons (RDS vs. no RDS) while fixing all other processing combinations. In each case, the use of RDS processing led to statistically lower (i.e., better) ratio test values. In fact, every mean ratio was lower when using RDS processing, often by a factor of 5–10. Further statistical analysis only considered processors using RDS noise correction.

We next statistically compared the six whitening variations separately for each combination of sampling rate and whitening band limit. Each Friedman test found a statistically significant difference. Nearly all ensuing paired comparisons were statistically different, with the exceptions listed in Table 3. All techniques performed well.

Thereafter, we statistically compared across sampling rates for each condition (i.e., across each row of Table 3) in the fashion described in Study 1. Significant differences were found in all paired tests. When whitening bandwidth was limited to 600 or 1000 Hz, the strength of these differences (comparing sampling rates of 4096–2048 Hz in Table 2) were not substantial. However, when whitening bandwidth was not limited (labeled “Nyquist” in Table 2), the trend was for the best performance at the 4096 Hz sampling rate and the worst performance at the 1024 Hz sampling rate.

Then, for subject-specific and IIR whiteners, we compared the performance of processors only using RDS processing vs. those only using ANC separately for each combination of sampling rate and whitening band limit. Each of these 14 differences was statistically significant. Only using RDS processing performed better than only using ANC in each case, except at the 1024 Hz sampling rate without bandwidth limiting.

## 5. Discussion

Though the investigation of efficient whitening computation (including the presence vs. absence of ANC and/or a band limit to the whitening) was the primary motivator for this research, we necessarily studied the associated roles of sampling rate and RDS noise correction. We also used two datasets in our investigation. The force-varying Study 1 data are representative of active muscle contractions, since they vary uniformly in effort over the range ±50% MVC and in frequency over the range of 0–1 Hz. Very limited low-level contraction segments are contained in such active data, but it is known that RDS processing is more relevant at lower contraction levels [26,52]. Thus, Study 2 used constant-force data from the lowest contraction level of 0% MVC (rest). The ratio between 0% and 50% MVC was computed because the use vs. absence of various processing stages can alter signal gain, making the relative size of noise vs. signal the relevant performance metric.

As anticipated by theory [26], offset correction via RDS processing was shown to be essential at lower contraction levels (e.g., the 0% MVCs of Study 2) but of limited value at higher contraction levels (Study 1). In Study 2, RDS processing combined with ANC and/or a bandwidth limit ≤1000 Hz produced a worst-case (i.e., highest) average ratio of 0.083—corresponding to an SNR of 12.0. For corresponding processors without RDS noise correction, this worst-case average ratio was 0.30—corresponding to an SNR of 3.3. Since low-effort contractions are common in many practical applications, RDS processing should be routinely included in EMG processors—the technique provides vital noise correction at low effort levels and does not reduce EMG processor performance at higher contraction levels. Furthermore, these results support the conclusion that RDS processing should be applied *even when* other methods (e.g., ANC, whitening bandwidth limiting) are used to reduce high frequency noise.

When both ANC and whitening filter band limiting were omitted at the 4096 Hz sampling rate, both the subject-specific and universal IIR whiteners performed poorer, on average, than unwhitened processing. Thus, these whitening options are not recommended. Since the EMG signal has a low-pass spectral shape, its SNR decreases with frequency. At some point in frequency, the true EMG signal power decreases below the noise floor. Because the EMG signal is amplitude-modulated by contraction effort, this “crossover” frequency increases with increasing contraction levels [27,34,35]. Whitening filters need to avoid amplifying these higher frequency regions. ANC does so by design and adjusts to the contraction level. Lower sampling rates and whitening filter band limiting do so in a more blunt manner. Likely, the high-pass and first difference whitening techniques do so by having lower high-frequency gain (see Figure 2).

Aside from these whitening methods in which high-frequency noise was not attenuated, the 2048 Hz sampling rate performed mildly poorer (~3%), in general, than the 4096 Hz rate. The 1024 Hz sampling rate, in general, performed modestly poorer still (~7%). These results are consistent with a prior EMG-force analysis of a subset of these data, suggesting that bandwidths out to 600–800 Hz are optimal [36]. A limitation of our work at different sampling rates is that we digitally decimated the original data samples from 4096 Hz down to 2048 and 1024 Hz. To avoid aliasing, we low-pass filtered the original samples at 80% of the new Nyquist rate prior to down-sampling. This commonly-used approach likely overly reduces signal magnitude at the highest frequencies of the decimated EMG signals. Whitening filters designed from these data, which are derived from the inverse of the magnitude spectrum, would have too high a gain at these frequencies. Furthermore, our second-order universal IIR whitening filters might also exhibit some gain distortion at lower frequencies, since low-order filters have limited degrees of freedom to follow shape changes. We did find, however, that higher-order IIR filters did not substantively reduce the mean squared error between the desired and the achieved magnitude responses.

Without whitening, the average Study 1 EMG-force error was 5.5% MVC. As shown in the results of Table 1, the absolute lowest average error (4.74% MVC) occurred when the data were sampled at 4096 Hz, with subject-specific whitening, no bandwidth limiting, using ANC, and RDS noise correction. This average error was lower than the corresponding error produced by the universal IIR whitening technique (4.83% MVC) but not statistically different. Given the large sample size (*N* = 64), this result calls into question the need to calibrate fixed whitening filters to each subject—at least for this range of contraction forces and conditions. The corresponding high-pass whitening technique exhibited an average error of 4.89% MVC, but these differences were again not statistically different. However, this method still requires the determination of the optimal high-pass cut-off frequency. Finally, the corresponding first difference method had average error performance that was comparable to that of the high-pass method (4.91% MVC) and also not statistically different from the others. Notably, the first difference whitening approach requires no calibration or algorithm decisions. It was surprisingly effective, even without the addition of a high-frequency noise attenuation stage. Because the “crossover” frequency at which noise power exceeds that of signal power is a function of the noise level of EMG recordings, lower noise causes the crossover frequency to move to higher frequencies. Accordingly, the location of bandwidth-limiting filters may need to change if the noise level changes significantly. For example, modern wireless EMG recording systems may admit more noise power in order to utilize lower-power electronics that extend battery life.

Though the evidence herein suggests that the fixed whitening filter (i.e., Stage 1 in Figure 3) might not need to be calibrated to each subject, we still calibrated ANC (i.e., Stages 2 and 3 in Figure 3) to the spectral shape of the noise measured from each subject. A fully universal whitening filter, including the removal of the need for any whitening calibration, is desired. Furthermore, the current work focused on the biceps and triceps muscles during modest contraction levels. It is not clear if the same universal IIR whitening filter is appropriate for other muscles or other contraction effort ranges.

## 6. Conclusions

While whitening has been known for several decades to improve the performance of features derived from the EMG signal (including EMGσ), its adoption has been limited, likely due to the complexity of its implementation. Thus, we compared four whitening methods (of various complexity levels) while varying the use of bandwidth-limiting techniques (ANC and whitening bandwidth limiting) at three different sampling rates and with vs. without RDS noise correction. We studied experimental data from 64 subjects contracting about the elbow by utilizing constant-posture, force-varying contractions over the range ± 50% MVC (Study 1) and constant-force contractions at 0% and 50% MVC (Study 2).

Regardless of other factors, our results found that RDS processing should be included whenever estimating EMGσ from the whitened EMG signal. Study 2 (which was designed to be most sensitive to additive EMG measurement noise) found a factor of up to 5–10 improvement in noise reduction when using RDS (vs. not using RDS) processing. In general, increasing the sampling rate provided small performance improvements. If EMG is sampled at 4096 Hz, then some form of high-frequency noise attenuation (ANC or direct bandwidth limitation) must be utilized when whitening—except that the simplified whitening techniques of high-pass filtering and first difference seem to have sufficiently low high-frequency gain so as to avoid this problem. The overall best performing variant (Nyquist bandwidth; including ANC, where relevant) at the 4096 Hz sampling rate were rank ordered (best to worst; Study 1) in average error as: subject-specific (4.74% MVC), universal IIR (4.83% MVC), high-pass (4.89% MVC), and first difference (4.91% MVC)—but none of these differences was statistically different. The first difference whitening method has a filter shape that is similar to a calibrated whitening filter for frequencies up to ~1000 Hz (Figure 2) but naturally lower gain thereafter. The lower high-frequency gain likely reduces high-frequency noise. The first difference technique also benefits from requiring no calibration and thus is an excellent option for general use, at least for this range of contraction forces and conditions.

## Figures and Tables

**Table 1:** Mean ± std. deviation of EMG-force errors (% MVC) for different whitening filter methods (*N* = 64), with RDS processing. Results shown for conditions: with vs. without use of adaptive noise canceling, different whitening bandwidth limits (“Nyquist” denotes no limiting used), and different sampling rates. For the high-pass whitening method, each result lists the optimal filter cut-off frequency. Note that, without any whitening or RDS processing, the mean ± standard deviation “baseline” EMG-force performance was 5.55 ± 2.4% MVC for all sampling rates.

Fixed Whiten Filter	Adaptive Noise Cancel?	White Band Limit	4096 Hz Sampling Rate	2048 Hz Sampling Rate	1024 Hz Sampling Rate
		600 Hz	4.90 ± 2.15	4.93 ± 2.18	—
	No	1000 Hz	5.00 ± 2.29	4.97 ± 2.30	—
Subject		Nyquist	6.03 ± 3.33	4.98 ± 2.30	5.05 ± 2.20
Specific		600 Hz	4.86 ± 2.06	4.93 ± 2.09	—
	Yes	1000 Hz	4.78 ± 2.10	4.85 ± 2.09	—
		Nyquist	4.74 ± 2.07	4.88 ± 2.09	5.08 ± 2.10
		600 Hz	4.95 ± 2.20	5.03 ± 2.18	—
	No	1000 Hz	4.84 ± 2.10	4.90 ± 2.10	—
IIR		Nyquist	5.57 ± 2.76	4.90 ± 2.09	5.08 ± 2.20
		600 Hz	4.95 ± 2.20	5.04 ± 2.17	—
	Yes	1000 Hz	4.79 ± 2.06	4.90 ± 2.09	—
		Nyquist	4.83 ± 2.07	4.91 ± 2.10	5.09 ± 2.22
		600 Hz	4.98 ± 2.15 (2047 Hz)	4.95 ± 2.15 (1023 Hz)	—
High-pass	N/A	1000 Hz	4.92 ± 2.10 (2047 Hz)	4.88 ± 2.10 (970 Hz)	—
		Nyquist	4.89 ± 2.06 (1300 Hz)	4.90 ± 2.11 (880 Hz)	4.98 ± 2.04 (490 Hz)
		600 Hz	5.00 ± 2.16	5.03 ± 2.22	—
First	N/A	1000 Hz	4.95 ± 2.12	5.00 ± 2.19	—
Diff.		Nyquist	4.91 ± 2.09	5.00 ± 2.18	5.12 ± 2.25

**Table 2:** Mean ± std. deviations of ratios of 0 % to 50% EMGσ for different fixed whitening filter methods (*N* = 64 subjects). Smaller ratios denote better performance.

Fixed Whiten Filter	Adaptive Noise Cancel?	White Band Limit	4096 Hz Sampling Rate		2048 Hz Sampling Rate		1024 Hz Sampling Rate	
			No RDS	Yes RDS	No RDS	Yes RDS	No RDS	Yes RDS
		600 Hz	0.13 ± 0.11	0.048 ± 0.11	0.13 ± 0.12	0.047 ± 0.11	—	—
	No	1000 Hz	0.26 ± 0.17	0.024 ± 0.097	0.23 ± 0.16	0.027 ± 0.099	—	—
Subject		Nyquist	0.55 ± 0.23	0.0073 ± 0.078	0.23 ± 0.16	0.027 ± 0.099	0.11 ± 0.11	0.056 ± 0.11
Specific		600 Hz	0.074 ± 0.076	0.048 ± 0.096	0.076 ± 0.087	0.048 ± 0.10	—	—
	Yes	1000 Hz	0.13 ± 0.11	0.041 ± 0.099	0.12 ± 0.11	0.042 ± 0.096	—	—
		Nyquist	0.34 ± 0.21	0.017 ± 0.090	0.12 ± 0.11	0.042 ± 0.095	0.076 ± 0.087	0.054 ± 0.098
		600 Hz	0.14 ± 0.12	0.054 ± 0.13	0.14 ± 0.14	0.059 ± 0.16	—	—
	No	1000 Hz	0.30 ± 0.19	0.024 ± 0.11	0.24 ± 0.19	0.033 ± 0.14	—	—
IIR		Nyquist	0.57 ± 0.22	0.0076 ± 0.082	0.24 ± 0.19	0.033 ± 0.14	0.12 ± 0.22	0.083 ± 0.34
		600 Hz	0.089 ± 0.082	0.066 ± 0.10	0.076 ± 0.093	0.060 ± 0.12	—	—
	Yes	1000 Hz	0.080 ± 0.083	0.059 ± 0.10	0.071 ± 0.091	0.055 ± 0.11	—	—
		Nyquist	0.070 ± 0.082	0.053 ± 0.096	0.071 ± 0.091	0.055 ± 0.11	0.078 ± 0.15	0.070 ± 0.24
		600 Hz	0.098 ± 0.092	0.051 ± 0.096	0.10 ± 0.095	0.051 ± 0.097	—	—
High-pass	N/A	1000 Hz	0.14 ± 0.11	0.043 ± 0.096	0.15 ± 0.12	0.041 ± 0.097	—	—
		Nyquist	0.23 ± 0.15	0.026 ± 0.089	0.14 ± 0.11	0.043 ± 0.097	0.098 ± 0.094	0.055 ± 0.10
		600 Hz	0.096 ± 0.091	0.051 ± 0.095	0.093 ± 0.090	0.052 ± 0.095	—	—
First	N/A	1000 Hz	0.13 ± 0.11	0.046 ± 0.096	0.11 ± 0.096	0.049 ± 0.095	—	—
Diff.		Nyquist	0.19 ± 0.13	0.032 ± 0.091	0.11 ± 0.096	0.049 ± 0.095	0.080 ± 0.084	0.052 ± 0.092

**Table 3:** Study 2 whitening method statistical results: this table lists non-significant paired comparisons between whitening methods after fixing the sampling rate and whitening band limit (RDS processing used). ANC = adaptive noise canceling. Sampling rate = *f_c_*.

*f_c_* (Hz)	Whitening Band (Hz)	Non-Significant Paired Comparisons
4096	600	IIR with ANC vs. (high-pass, first difference)
4096	1000	Subject-specific without ANC vs. IIR without ANC; IIR with ANC vs. high-pass
4096	Nyquist	—
2048	600	Subject-specific without ANC vs. (IIR with ANC, high-pass); IIR with ANC vs. (high-pass, first difference)
2048	1000	Subject-specific without ANC vs. IIR without ANC
2048	Nyquist	Subject-specific without ANC vs. IIR without ANC
1024	Nyquist	Subject-specific without ANC vs. IIR with ANC; high-pass vs. first difference

## Data Availability

No new data were collected during this study.

## Appendix A

A universal first-stage IIR whitening filter was separately designed for each utilized sampling rate (4096, 2048, and 1024 Hz). Each filter can be realized as a linear constant-coefficient difference equation. These difference equations, with input x[n] and output y[n], where n is the sample index, are given in Table A1 for these frequencies and for the commonly-used sampling frequencies of 4000, 2000, and 1000 Hz.

**Table A1:** Equations defining the universal first-stage IIR whitening filter at various sampling rates.

Sample Rate (Hz)	Universal IIR Whitening Filter, Defined as a Linear, Constant-Coefficient Difference Equation: $y\left[n\right]=b_{0}\;x\left[n\right]+b_{1}\;x\left[n-1\right]+b_{2}\;x\left[n-2\right]-a_{1}\;y\left[n-1\right]-a_{2}\;y\left[n-2\right]$				
	$b_{0}$	$b_{1}$	$b_{2}$	$a_{1}$	$a_{2}$
1000	–5.10427	6.82006	–4.09619	0.742714	–0.128509
1024	–3.90799	5.90018	–4.23552	0.800134	–0.0871683
2000	–6.81618	12.9140	–7.89417	0.632655	–0.136978
2048	–7.20675	13.2972	–7.80079	0.760178	–0.00269560
4000	–17.5275	32.1657	–15.3385	0.452029	0.0876669
4096	–17.5038	31.2572	–14.6111	0.371506	0.0980280
